# Postoperative serum squamous cell carcinoma antigen and carcinoembryonic antigen predict overall survival in surgical patients with esophageal squamous cell carcinoma

**DOI:** 10.3389/fonc.2023.1263990

**Published:** 2023-09-22

**Authors:** Yi Huang, Fangfang Liu, Ruiping Xu, Fuyou Zhou, Wenlei Yang, Yu He, Zhen Liu, Bolin Hou, Linlin Liang, Lixin Zhang, Mengfei Liu, Yaqi Pan, Ying Liu, Zhonghu He, Yang Ke

**Affiliations:** ^1^ State Key Laboratory of Molecular Oncology, Beijing Key Laboratory of Carcinogenesis and Translational Research, Department of Genetics, Peking University Cancer Hospital & Institute, Beijing, China; ^2^ Anyang Cancer Hospital, Henan, China; ^3^ Chinese Preventive Medicine Association, Beijing, China; ^4^ Linkdoc AI Research (LAIR), Beijing, China

**Keywords:** esophageal squamous cell carcinoma, prognosis, postoperative tumor marker, squamous cell carcinoma antigen, carcinoembryonic antigen

## Abstract

**Background:**

Tumor markers are routinely used in clinical practice. However, for resectable patients with esophageal squamous cell carcinoma (ESCC), they are applied infrequently as their prognostic significance is incompletely understood.

**Methods:**

This historical cohort study included 2769 patients with resected ESCC from 2011 to 2018 in a high-risk area in northern China. Their clinical data were extracted from the Electronic Medical Record. Survival analysis of eight common tumor markers was performed with multivariable Cox proportional hazards regressions.

**Results:**

With a median follow-up of 39.5 months, 901 deaths occurred. Among the eight target markers, elevated postoperative serum SCC (Squamous cell carcinoma antigen) and CEA (Carcinoembryonic antigen) predicted poor overall survival (SCC HR_adjusted_: 2.67, 95% CI: 1.70-4.17; CEA HR_adjusted_: 2.36, 95% CI: 1.14-4.86). In contrast, preoperative levels were not significantly associated with survival. Stratified analysis also demonstrated poorer survival in seropositive groups of postoperative SCC and CEA within each TNM stage. The above associations were generally robust using different quantiles of concentrations above the upper limit of the clinical normal range as alternative cutoffs. Regarding temporal trends of serum levels, SCC and CEA were similar. Their concentrations fell rapidly after surgery and thereafter remained relatively stable.

**Conclusion:**

Postoperative serum SCC and CEA levels predict the overall survival of ESCC surgical patients. More importance should be attached to the use of these markers in clinical applications.

## 1 Introduction

Esophageal cancer is currently ranked 7^th^ and 6^th^ worldwide in incidence and mortality respectively (1). Esophageal cancer has two major histologic types: esophageal adenocarcinoma (EAC) and esophageal squamous cell carcinoma (ESCC) (2), which account for 90% of patients in China (3). Esophageal squamous cell carcinoma has a poor prognosis, with overall survival of about 20% at 5 years (4). Surgical ESCC patients have a 40%-59% 5-year survival (4, 5).

Current treatment of esophageal cancer is tailored to TNM stage in order to ensure the best clinical outcomes (6). However, the prognosis is heterogeneous even in ESCC patients of identical stage (7). Evaluation and integration of additional significant prognostic factors, such as serum tumor marker levels, may yield a more accurate estimate of ESCC prognosis (8).

Tumor markers are substances released by cancer cells or produced by other cells of the body in response to a malignant tumor or benign condition (9). Tumor markers allow convenient non-invasive assessment of prognosis. Associations between certain perioperative serum tumor markers and prognosis in certain cancers were consistently confirmed. Thus, such markers are used frequently in clinical practice as for example, CEA (Carcinoembryonic antigen) in colon tumors (10, 11), CA19-9 (Carbohydrate antigen 19-9) in pancreatic cancer (12) and a combination of AFP (Alpha-fetoprotein) and HCG (Human chorionic gonadotropin) in testicular germ-cell tumors (13). Regarding current evidence on relationships between tumor markers and prognosis of ESCC, no consensus was reached. Some studies have shown that elevated tumor markers portend a poorer prognosis (14–17), while other markers have shown no significant relationships with prognosis (18–20). The limitations of most previous studies have largely restricted their clinical application in stratification of prognosis. Of note, almost all prior studies of ESCC have focused on preoperative tumor markers and lacked real-world postoperative evaluation. Moreover, there were limitations due to a series of flaws in study design including 1) small sample size, 2) inadequate control for confounding factors, and 3) short-term follow-up.

In this study, based on a large real-world clinical cohort with up to 7 years of follow-up, we aimed to systematically evaluate associations of common tumor markers and overall survival of ESCC patients undergoing radical resection, both preoperatively and postoperatively, and to provide compelling evidence to promote proper clinical application of tumor markers in ESCC surgical patients.

## 2 Materials and methods

### 2.1 Study patients

This study was conducted at the Anyang Cancer hospital (Anyang city, Henan province, China), which is the only tertiary specialized cancer hospital in northern Henan province. This area is a traditional high-risk area for ESCC (21). We consecutively recruited 2,789 ESCC patients undergoing radical resection at this hospital from November 2011 to July 2018, who had information about at least one of eight target tumor markers and more than 6 months of follow-up. We excluded patients who (1) were diagnosed with other cancers at admission (2) died during the hospital stay or within 30 days after surgery, (3) had metastatic disease (TNM Stage M1), or (4) received endoscopic therapy.

### 2.2 Data collection and processing

All of the study patient data were extracted from the Electronic Medical Record (EMR). This included covering baseline demographic factors, tumor characteristics, treatment data, and serum tumor markers which had been tested in the laboratory. TNM stage was classified according to the 7th edition of the American Joint Committee on Cancer (AJCC)/International Union for Cancer Control (UICC) TNM system.

### 2.3 Survival follow-up

The endpoint of our study was overall survival (OS), defined as the interval from the date of the first admission of a given patient to death from any cause, or the last follow-up. The date of the first admission was, on average, 11.7 (standard deviation [SD]:17.32) days before the surgery date. As described previously (8), all patient follow-up was carried out through outpatient visits or telephone interviews. The last follow-up date was July 19, 2018. Follow-up rate was defined as the proportion of all patients who died or had at least one follow-up record. This comprised 76.8% of the patients in our study. The median follow-up time was 39.5 months (interquartile range [IQR]: 38.5-40.2 months).

### 2.4 The detection and sample size for each tumor marker

In line with the clinical protocol, a 3-milliliter venous blood specimen was collected. This specimen was subsequently centrifuged at 3,000 rpm for 10 minutes at ambient temperature. Following this, the processed specimen underwent precise immunoassay analysis using the AutoLumo A2000 Plus and Beckman DxI 800 instruments, both of which were fully automated chemiluminescence immunoassay systems

Eight common tumor markers including CA125 (Carbohydrate antigen 125), CA15-3 (Carbohydrate antigen 15-3), CA724 (Carbohydrate antigen 724), CEA (Carcinoembryonic antigen), CYFRA21-1 (Cytokeratin 19 fragment), NSE (Neuron-Specific enolase), SCC (Squamous cell carcinoma antigen), and TSGF (Tumor specific growth factor) were detected.

The utilization of tumor markers for ESCC varied greatly based on the individual experience of the clinicians, even in the case of multiple tests conducted on the same patient. As a result, the panel of tumor markers tested was not consistent among all patients, leading to the different sample sizes for each tumor marker. Additionally, some patients underwent preoperative testing only, while others underwent postoperative testing only. Therefore, the preoperative and postoperative samples for the same tumor marker were inconsistent.

Serum levels of each tumor marker were dichotomized by the recommended upper limit of the normal range used in clinical practice, and the higher levels were defined as seropositive. Preoperative seropositivity of tumor markers was defined by utilizing the latest serum levels within 14 days before radical resection, to capture the most recent stable measurements. Postoperative seropositivity was defined based on the first detection results within 14 to 180 days after radical resection, aiming to minimize the variability in tumor marker levels potentially caused by surgical trauma.

### 2.5 Statistical analysis

To avoid potential technical errors, we excluded tumor markers with serum concentrations beyond 5 standard deviations (SDs) from the mean (2 [0.7%] outliers). Kaplan-Meier survival curves for overall survival were delineated and the log-rank test was used to compare positive and negative marker levels. Cox proportional hazards regression was employed to evaluate the associations between tumor markers and prognosis, adjusting for known prognostic factors and other potential confounders including age, sex, tumor site, TNM stage, number of lymph nodes harvested, tumor size, surgical margin status, preoperative neoadjuvant treatment and postoperative adjuvant treatment.

To explore the temporal trends of certain tumor markers, time intervals from the date of tumor marker testing to the date of surgery were grouped into 0-7, 7-14, 14-30 days preoperatively and 0-7,7-14, 14-30, 30-60, 60-90, 90-120, 120-150, 150-180, 180-360 days postoperatively. Smoothed trend curves were delineated by calculating the median of tumor markers’ concentrations tested at different time intervals.

We also performed three sets of sensitivity analyses to evaluate the robustness of our prognostic analysis by 1) adopting different quantiles of tumor markers as cutoffs, stepping by 0.01 from 0.05 to 0.99; 2) using alternative time windows to define included cases for each perioperative analysis (preoperatively: tumor markers were tested within 7, 14, or 30 days before surgery; postoperatively: markers were tested within 30, 60, 90 150, 180, or 360 days after surgery); 3) and by excluding “peri-testing” dead cases (e.g., died within 6 months of the first measurement of serum tumor markers after surgery) to assess the long-term prognostic value of tumor markers. All curves were smoothed by the locally weighted scatterplot smoothing (LOWESS) method.

Statistical analyses were conducted using R software (version 4.1.2, Mac). The “readstata13” package facilitated data cleaning, while the “survival”, “survminer”, “pwr”, and “table1” packages supported our data analysis. Visualization was accomplished with the “ggplot2” package. Two-sided P values < 0.05 were considered statistically significant. And this work has been reported in line with the STROCSS criteria (22).

### 2.6 Ethics statement

This study was approved by the Institutional Review Board of the Peking University School of Oncology, China (the relevant Judgement’s reference number: 2018KT68).

## 3 Results

### 3.1 Patient characteristics

A total of 2,769 ESCC patients were finally included in this study. 45.1% of patients were aged 65 or older, and 62.3% were male (**Table 1**). Among the patients, 45.4% had a history of smoking, while 35.5% had a history of alcohol use. Almost two-thirds of patients (64.0%) were classified as stage I-II. The majority of tumors (64.0%) were located in the middle part of the esophagus, contrasting with 15.9% in the upper esophagus, and 19.0% in the lower. The mean tumor size was 4.0 cm (IQR: 3.0-5.0 cm). 55.4% of these patients received surgery alone, and 44.6% received additional postoperative adjuvant therapy. Only 10.7% of patients received preoperative neoadjuvant therapy. A median of 15 lymph nodes (IQR: 11-20 lymph nodes) were harvested from patients.

**Table 1:** Baseline clinicopathologic characteristics of ESCC patients undergoing the radical resection from Anyang Cancer Hospital, 2011-2018.

Clinicopathologic characteristics		Total (N=2769)	Death (N=901)
		N (%)	N (%)
Age (years)			
	<65	1522 (54.9)	458 (50.8)
	≥65	1247 (45.1)	443 (49.2)
Sex			
	Female	1044 (37.7)	310 (34.4)
	Male	1725 (62.3)	591 (65.6)
Smoking history			
	No	1405 (50.7)	445 (49.4)
	Yes	1257 (45.4)	423 (46.9)
Alcohol-use history			
	No	1648 (59.5)	539 (59.8)
	Yes	986 (35.5)	323 (35.8)
Tumor site			
	Upper	441 (15.9)	185 (20.5)
	Middle	1773 (64.0)	545 (60.5)
	Lower	527 (19.0)	168 (18.6)
TNM stage^a^			
	I-II	1784 (64.0)	422 (46.6)
	III	990 (35.5)	483 (53.3)
Number of lymph nodes harvested			
	<20	2005 (71.5)	680 (75.4)
	≥20	742 (26.8)	211 (24.5)
Tumor size (cm)			
	Median [IQR]	4.0 [3.0, 5.0]	4.5 [3.5, 5.0]
Surgical margins status			
	Negative	2726 (98.5)	875 (97.8)
	Positive	42 (1.5)	20 (2.2)
Preoperative neoadjuvant treatment			
	No	2473 (89.3)	786 (87.2)
	Yes	296 (10.7)	115 (12.8)
Postoperative adjuvant treatment			
	No (Surgery only)	1533 (55.4)	427 (47.4)
	CT	1012 (36.5)	363 (40.3)
	RT	96 (3.5)	45 (5.0)
	CRT	128 (4.6)	66 (7.4)

CRT, surgery and adjuvant chemoradiotherapy; CT, surgery and adjuvant chemotherapy; ESCC, esophageal squamous cell carcinoma; IQR, interquartile range; N, number of patients available; RT, surgery and adjuvant radiotherapy; TNM, tumor node metastasis.

a Some clinicopathologic data were missing, such as T stage.

### 3.2 Associations between tumor markers and overall survival

Using the upper limit of the clinical normal range as cutoffs, preoperative seropositivity of tumor markers ranged from 2.0%-31.5%, and postoperative seropositivity ranged from 0.0%-27.2%. In multivariable analysis, there was no significant association of preoperative serum levels and overall survival observed for any of the eight tumor markers included. In contrast, elevated postoperative serum SCC and CEA predicted poor overall survival (SCC HR_adjusted_: 2.67, 95% CI: 1.70-4.17; CEA HR_adjusted_: 2.36, 95% CI: 1.14-4.86) (**Table 2**). When both serum SCC and CEA were incorporated into the multivariable analysis, postoperative SCC remained significantly associated with prognosis, whereas the postoperative serum CEA and prognosis demonstrated a weak association. Similarly, no significant correlations were found between prognosis and preoperative SCC and CEA (**Table S7**, **S8**). Stratified analysis showed similar findings within each stage (**Table S2**).

**Table 2:** Relationship between tumor markers and overall survival in ESCC patients receiving radical resection using univariable and multivariable Cox proportional hazards regression model.

			Preoperative (within 14 days)^b^			Postoperative (within 14-180 days)^c^	
Tumor markers^a^		N (%)	Crude HR (95% CI)	Adjusted HR (95% CI)^d^	N (%)	Crude HR (95% CI)	Adjusted HR (95% CI)^d^
CA125							
Negative (< 33 U/mL)		389 (98.0)	Ref.	Ref.	67 (72.8)	Ref.	Ref.
Positive (≥ 33 U/mL)		8 (2.0)	0.64 (0.09-4.6)	0.94 (0.6-1.46)	25 (27.2)	1.80 (0.85-3.78)	1.37 (0.64-2.91)
CA15-3							
Negative (< 25 U/mL)		170 (96.6)	Ref.		15 (100.0)		
Positive (≥ 25 U/mL)		6 (3.4)	0.77 (0.11-5.59)	NA	0 (0.0)	NA	NA
CA724							
Negative (< 6 IU/mL)		165 (90.3)	Ref.	Ref.	382 (85.7)	Ref.	Ref.
Positive (≥ 6 IU/mL)		18 (9.8)	0.82 (0.25-2.63)	1.87 (0.43-8.18)	64 (14.3)	1.31 (0.87-1.97)	1.01 (0.52-1.97)
CEA							
Negative (< 5 ng/mL)		1135 (96.4)	Ref.	Ref.	549 (97.0)	Ref.	Ref.
Positive (≥ 5 ng/mL)		43 (3.7)	1.26 (0.72-2.19)	1.30 (0.69-2.46)	17 (3.0)	3.68 (1.94-6.96)	2.36 (1.14-4.86)
CYFRA21-1							
Negative (< 7 ng/mL)		177 (95.7)	Ref.		145 (92.9)	Ref.	Ref.
Positive (≥ 7 ng/mL)		8 (4.3)	2.64 (0.94-7.46)	NA	11 (7.1)	1.89 (0.75-4.77)	0.92 (0.21-4.06)
NSE							
Negative (< 10 ng/mL)		116 (90.6)	Ref.		17 (94.4)		
Positive (≥ 10 ng/mL)		12 (9.4)	0.67 (0.20-2.20)	NA	1 (5.6)	NA	NA
SCC							
Negative (< 1.5 ng/mL)		359 (68.7)	Ref.	Ref.	261 (80.4)	Ref.	Ref.
Positive (≥ 1.5 ng/mL)		165 (31.5)	1.61 (1.12-2.32)	1.32 (0.89-1.95)	64 (19.7)	3.07 (2.04-4.61)	2.67 (1.70-4.17)
TSGF							
Negative (< 64 U/mL)		398 (87.6)	Ref.	Ref.	129 (74.7)	Ref.	Ref.
Positive (≥ 64 U/mL)		57 (12.5)	1.18 (0.76-1.83)	1.14 (0.70-1.87)	44 (25.4)	1.36 (0.78-2.39)	1.20 (0.63-2.29)

We also evaluated the prognostic significance of the combination of SCC and CEA on survival. Patients with both positive SCC and CEA tended to have poorer survival, compared with those with negative serum levels for either or both SCC and CEA (**Table S1**).

**Figure 1:**
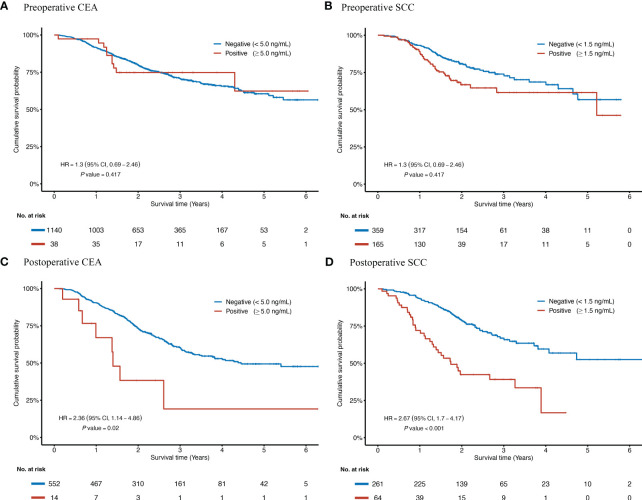
Kaplan-Meier curves according to the status of preoperative and postoperative CEA and SCC for ESCC patients receiving radical resection. HRs and *P* values were adjusted for a set of fixed confounders including age, sex, smoking history, alcohol-use history, tumor site, TNM stage, number of lymph nodes harvested, tumor size, surgical margin status, preoperative neoadjuvant treatment, and postoperative adjuvant treatment. Kaplan-Meier curves of overall survival are based on the preoperative concentration of CEA **(A)**, the preoperative concentration of SCC **(B)**, the postoperative concentration of CEA **(C)**, and the postoperative concentration of SCC **(D)**. CEA, Carcinoembryonic antigen; CI, confidence interval; HR, hazard ratio; ESCC, esophageal squamous cell carcinoma; No., Number of the patients; SCC, Squamous cell carcinoma antigen.

The Kaplan-Meier curves for postoperative tumor markers showed that seropositivity of either SCC (**Figure 1C**, log-rank *P* < 0.001) or CEA (**Figure 1D**, log-rank *P* = 0.003) yielded poorer survival. However, curves for preoperative CEA (**Figure 1A**) and SCC (**Figure 1B**) were indistinguishable.

### 3.3 Temporal trends of SCC and CEA concentrations

**Figure 2:**
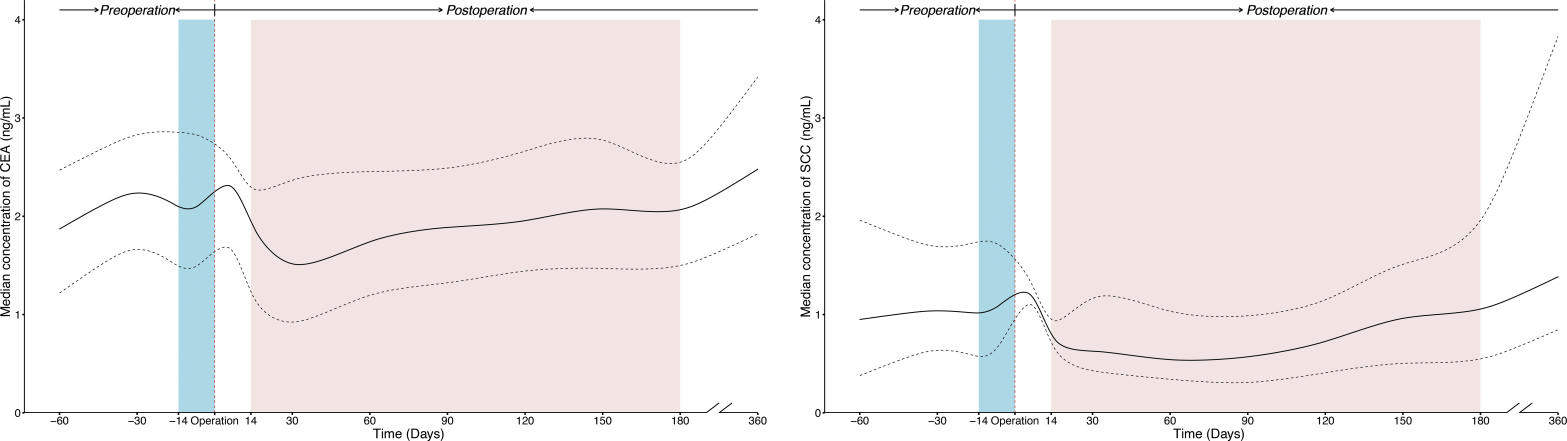
The temporal trends of perioperative concentration for SCC and CEA in ESCC patients receiving radical resection. The solid line was plotted using the median concentration of tumor markers tested in each time window, and the dotted line was plotted using the interquartile ranges of the concentration. The blue and red shaded areas present preoperative and postoperative time windows applied in prognostic analysis respectively. All curves were smoothed by the locally weighted scatterplot smoothing method. CEA, Carcinoembryonic antigen; CI, confidence interval; ESCC, esophageal squamous cell carcinoma; SCC, Squamous cell carcinoma antigen.

The temporal trends of SCC and CEA concentrations were similar, as shown in **Figure 2**. Before surgery, the serum levels of tumor markers were stable. After surgery, the serum levels of tumor markers fell rapidly from 0 to 14 (or 30) days and remained relatively stable from thereafter till 180 days.

### 3.4 Sensitivity analysis

**Figure 3:**
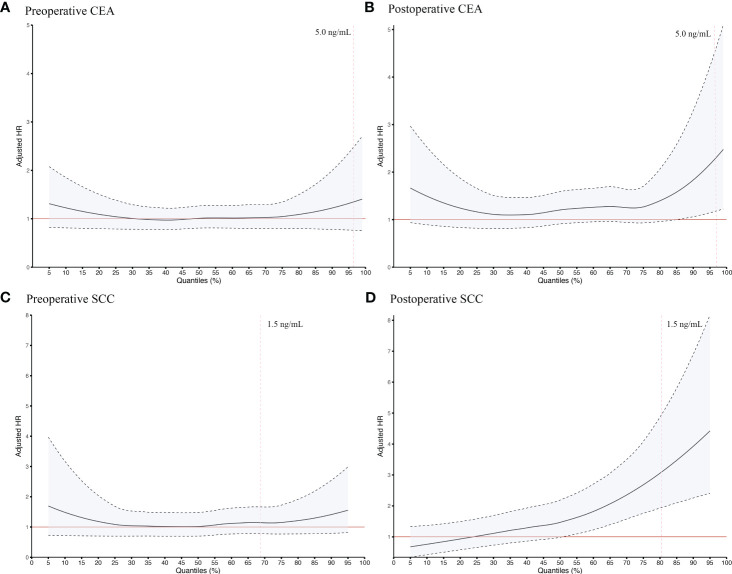
Sensitive survival analysis of preoperative CEA **(A)**, postoperative CEA **(B)**, preoperative SCC **(C)** and postoperative SCC **(D)** using different quantiles as cutoff points in ESCC patients undergoing radical resection. The quantiles stepped by 1% were taken as the cutoff value to distinguish Negative and Positive groups and to calculate the HRs by adjusting for a set of fixed confounders including age, sex, smoking history, alcohol-use history, tumor site, TNM stage, number of lymph nodes harvested, tumor size, surgical margin status, preoperative neoadjuvant treatment, and postoperative adjuvant treatment. All curves were smoothed by the locally weighted scatterplot smoothing method. The red line, adjusted HR = 1; the black dotted line, 95% confidence interval of the adjusted HR; the vertical red dotted line, the recommended upper limit of the normal range commonly applied in clinical practice for defining seropositivity of tumor markers. CEA, Carcinoembryonic antigen; ESCC, esophageal squamous cell carcinoma; HR, hazard ratio; SCC, Squamous cell carcinoma antigen.

In order to verify the robustness of our main results, different cutoffs were used to repeatedly divide study subjects into Positive/Negative groups. Adjusted HRs for SCC and CEA were generally robust at concentrations above the upper limit of the clinical normal range (1.5 ng/mL for SCC and 5.0 ng/ml for CEA), as shown in **Figure 3**. When different time windows were used to determine which cases were included for perioperative analysis, the adjusted HRs for SCC and CEA remained consistent across time windows (data not shown).

Moreover, after excluding cases who had died within 6 months of the first measurement of serum tumor markers after surgery, elevated postoperative serum SCC and CEA still predicted poor overall survival (SCC HR_adjusted_: 2.56, 95% CI: 1.60-4.10, CEA HR_adjusted_: 2.74, 95% CI: 1.34-5.63).

## 4 Discussion

The TNM staging system has been accepted as the most important prognostic predictor in ESCC patients. Nevertheless, the prognosis of ESCC still may be heterogeneous among ESCC patients with identical stage tumors (7, 8). Tumor markers with independent prognostic significance may be valuable as adjuncts for the TNM staging system. To date, tumor markers have been routinely used to help evaluate prognosis in clinical practice of certain cancers (23). However, due to lack of compelling evidence regarding the clinical significance of these markers, the pattern of current tumor marker use for ESCC is largely dependent on the personal experience of the clinicians. In an attempt to more exactly predict patient outcomes and promote the clinical utility of tumor markers in ESCC, we for the first time, systematically evaluated the associations of pre- and post-operative seropositivity of eight tumor markers and overall survival in a real-world setting based on the 2789 resectable ESCC patients with up to 7 years of follow-up. This demonstrates that postoperative SCC and CEA are independent prognostic factors for survival and may be used to inform clinical decisions.

SCC is a subfraction of the tumor antigen TA-4, which was originally purified from the human uterine cervix by Kato and Torigoe (24). An amino acid homology search for this molecule showed SCC is a member of the serine protease inhibitor family (25), which serves to modulate angiogenesis and support tumor growth and progression (26). Previous studies have shown that SCC plays a role in the prevention of TNF--induced cell death (27). Our results corroborate the correlation of postoperative serum SCC with overall survival and may be of use in distinguishing high-risk groups in ESCC patients. Few studies have focused on the prognostic value of postoperative SCC in ESCC. Despite the small sample size, findings of H Shimada et al. were similar to ours (17). Evaluation of other cancers has also demonstrated that postoperative SCC has a significant role in determining prognosis. For example, in cervical squamous cell carcinoma, elevated posttreatment serum SCC has been considered to be a risk factor for cancer recurrence after complete remission (28), and for survival using SCC cutoffs ranging from 1.5 to 2.0 ng/mL (29).

CEA was first isolated from human colon cancer tissue in 1965 by Gold and Freedman (30, 31). The gene sequencing suggests that CEA may act as an adhesion molecule, and CEA does seem to play a role in invasion and metastasis (32). Although the prognostic significance of postoperative CEA has not previously been evaluated in ESCC, studies of colon cancer have repeatedly demonstrated that decreasing postoperative serum CEA has independent predictive value for better overall survival (11, 33, 34).

In contrast with postoperative seropositivity, preoperative seropositivity of any of the eight targeted tumor markers showed no significant relationship with patients’ survival. Notably, although univariable analysis showed preoperative SCC has a significant relationship with prognosis, the multivariable analysis yielded only negative results, in agreement with a number of previous studies (19, 20, 35, 36). There have also been findings to the contrary (14, 16, 17), but the majority of these studies had methodological issues such as inadequate adjustment for confounding factors which may have caused apparent associations of preoperative SCC concentration and prognosis which were in fact spurious. Most previous studies have shown that the preoperative level of CEA is not a significant independent predictor of prognosis (17–19, 37). Although Yang et al. (20) reported that preoperative CEA may be a prognostic indicator for ESCC, it is very likely their findings were confounded as only the log-rank test was employed instead of multivariable modeling analysis.

The discrepancy between pre- and post-operative tumor markers’ prognostic significance may be explained partially by tumor burden. It has been proposed that the clearance rate of tumor markers after treatment may have prognostic significance (38). If a tumor is completely extirpated by surgery, there is no source of tumor marker production and ideal serum tumor markers, which reflect the total amount of cancer in the body would be negative (39). Positive postoperative results for serum tumor markers may reflect the presence of occult residual diseases and have potential to bring about subsequent biologic disorders. As seen in colon cancer, elevated postoperative CEA rather than elevated preoperative CEA normalized after resection, and this was an indicator of poor prognosis (11). With regard to prognosis for ESCC, the postoperative serum tumor markers seem to provide more tumor burden-related information than the preoperative markers.

To evaluate the temporal trend and determine the optimal time window for prognostic analysis, we plotted serum concentration-time curves for SCC and CEA based on population-level data. Serum levels of SCC and CEA declined after tumor resection, which was consistent with the individual disappearance curves of tumor markers as seen elsewhere (40). Multiple studies have reported a logarithmic decrease in the disappearance curves of tumor markers after tumor resection (38, 40, 41). For SCC and CEA, decreases in concentration curves (0-14 days) mirroring the clearance of serum tumor markers (41) may reflect a decrease in cancer burden. Based on the temporal trends, our study performed prognostic analysis using a stable period (14-180 days) and excluding a decreasing time interval (0-14 days), to ensure the stability of the association between the postoperative serum tumor markers and overall survival.

The limited evidence regarding prognostic relevance of tumor markers poses challenges for applying tumor markers in routine clinical examination for ESCC patients. Tumor markers have not been implemented in routine clinical management even in some tertiary hospitals in China. In this study, owing to the availability of large-scale, real-world clinical EMR data, we 1) had relatively adequate statistical power to assess targeted markers both pre- and post-operatively (*post-hoc* test: >95% power for SCC and CEA); 2) and we were able to adjust up to eleven conventional confounding factors which ensured the establishment of an independent association between serum tumor markers and prognosis in ESCC patients. Consequently, the compelling evidence provided by our study may maximize the likelihood of the routine use of tumor markers in clinical practice. Also, our study provides implications for patient management. First, early intervention becomes paramount for patients with postoperative seropositivity of SCC and CEA, allowing clinicians to schedule more frequent follow-ups or initiate other therapies. Additionally, the postoperative serum level of SCC and CEA can be instrumental in risk assessment, helping to stratify patients for graded surveillance, and ensuring high-risk patients receive the necessary support and services. Moreover, our study adopted the clinically recommended cutoffs for SCC (1.5 ng/mL) and CEA (5 ng/mL) to define seropositivity, which may further facilitate their clinical usage.

Our study has limitations. This is a historical cohort study that relied exclusively on single-institutional data. Validation in other populations and geographic areas is needed. Despite the large sample size of the whole cohort, some subgroups of the eight targeted tumor markers were still relatively small due to their infrequent utilization in clinical practice, and this may have limited the statistical power in subgroup analysis.

In summary, postoperative serum SCC and CEA are independent factors for prognosis in ESCC surgical patients. We recommend routine use of SCC and CEA to inform clinical decisions directly. Furthermore, they are also candidates for use as predictors in statistical models that predict individualized survival for ESCC surgical patients (8).

## Data availability statement

The raw data supporting the conclusions of this article will be made available by the authors, without undue reservation.

## Ethics statement

The studies involving humans were approved by the Institutional Review Board of the Peking University School of Oncology, China (2018KT68). The studies were conducted in accordance with the local legislation and institutional requirements. Written informed consent for participation was not required from the participants or the participants’ legal guardians/next of kin in accordance with the national legislation and institutional requirements.

## Author contributions

YiH: Data curation, Formal Analysis, Methodology, Software, Visualization, Writing – original draft. FL: Data curation, Formal Analysis, Methodology, Visualization, Writing – review & editing. RX: Resources, Writing – review & editing. FZ: Resources, Writing – review & editing. WY: Data curation, Methodology, Writing – review & editing. YuH: Data curation, Methodology, Writing – review & editing. ZL: Methodology, Formal Analysis, Writing – review & editing. BH: Data curation, Writing – review & editing. LL: Methodology, Formal Analysis, Writing – review & editing. LZ: Data curation, Writing – review & editing. ML: Methodology, Writing – review & editing. YP: Methodology, Writing – review & editing. YL: Methodology, Writing – review & editing. ZH: Conceptualization, Methodology, Resources, Supervision, Writing – review & editing. YK: Conceptualization, Supervision, Writing – review & editing, Methodology.
