# Supplementary material for: Postoperative serum squamous cell carcinoma antigen and carcinoembryonic antigen predict overall survival in surgical patients with esophageal squamous cell carcinoma
Source: Front Oncol. 2023 Sep 22;13:1263990. doi: 10.3389/fonc.2023.1263990 (PMC10556684; doi:10.3389/fonc.2023.1263990)
Supplement: Supplementary file 1 [file DataSheet_1.docx]

Supplementary Material

# Supplementary Figures and Tables

This appendix has been provided by the authors to give readers additional information about their work.

## Supplementary Figures

**Supplementary Figure 1.** **Kaplan-Meier curves by risk groups based on preoperative/postoperative tumor markers for all ESCC patients receiving radical resection.**


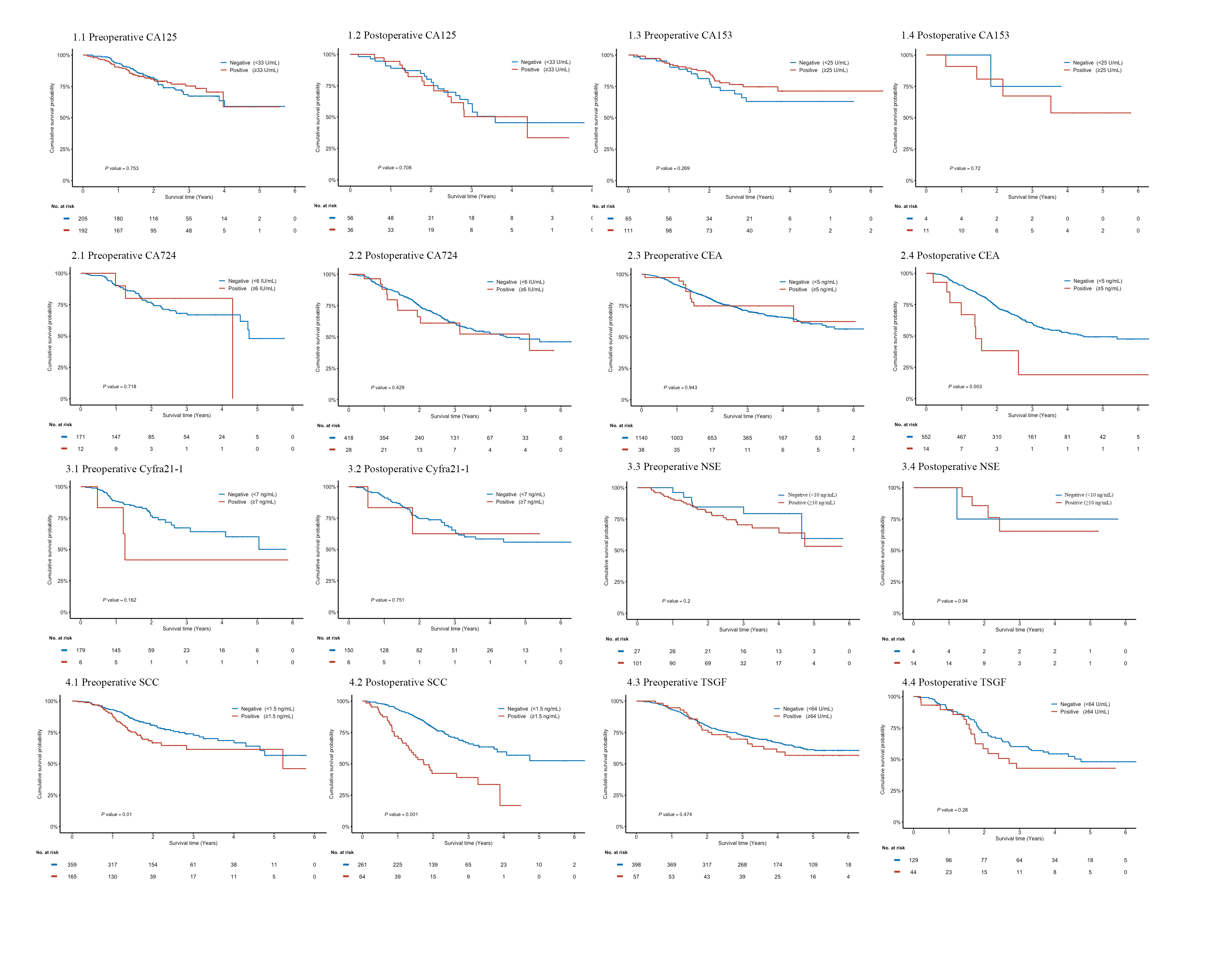


*P* values were calculated by log-rank test. **Abbreviations:** CA125, Carbohydrate antigen 125; CA15-3, Carbohydrate antigen 15-3; CA724, Carbohydrate antigen 724; CEA, Carcinoembryonic antigen; CI, confidence interval; CYFRA21-1, Cytokeratin 19 fragment; ESCC, esophageal squamous cell carcinoma; NSE, Neuron-Specific enolase; SCC, Squamous cell carcinoma associated antigen; TSGF, Tumor supplied group of factors

**Supplementary Figure 2. the correlation plot of eight tumor markers.**
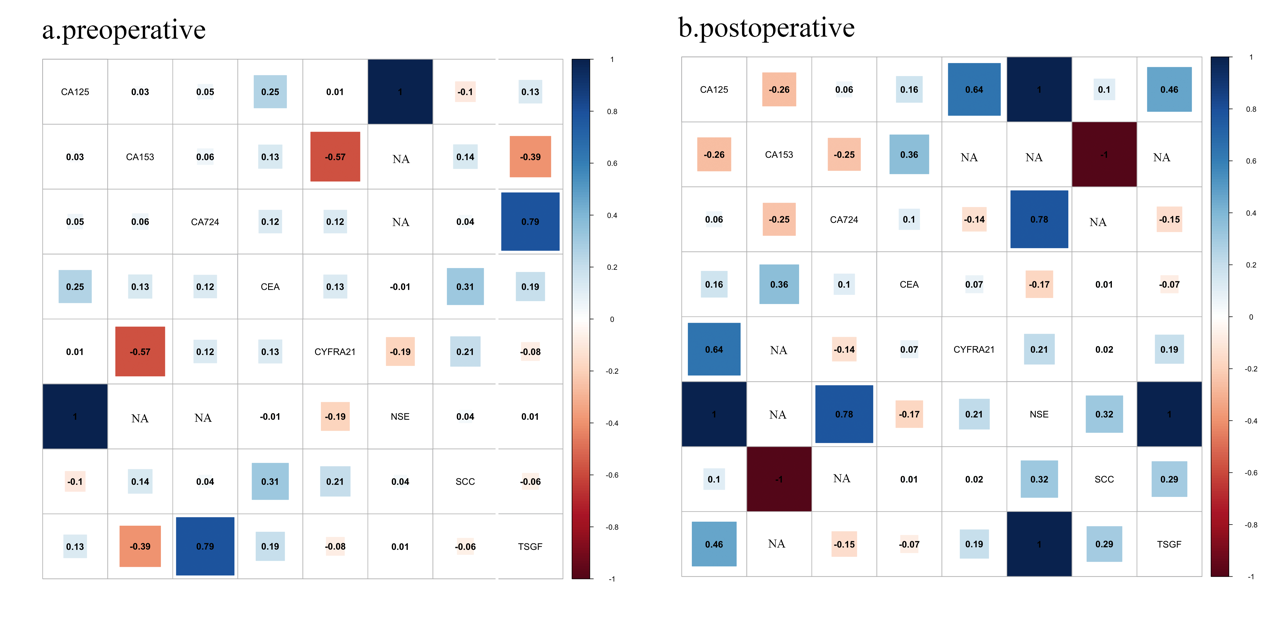


"The correlation coefficient values were calculated using Pearson's correlation. **Abbreviations:** NA: data not available (no subjects simultaneously detected the two tumor markers); CA125, Carbohydrate antigen 125; CA15-3, Carbohydrate antigen 15-3; CA724, Carbohydrate antigen 724; CEA, Carcinoembryonic antigen; CYFRA21-1, Cytokeratin 19 fragment; ESCC, esophageal squamous cell carcinoma; NSE, Neuron-Specific enolase; SCC, Squamous cell carcinoma associated antigen; TSGF, Tumor supplied group of factors

## Supplementary Tables

| Supplementary Table 1. Evaluation of prognostic significance for the indicator combining CEA and SCC by Cox proportional hazards regression model | | | | | | | |
| --- | --- | --- | --- | --- | --- | --- | --- |
|  | **Preoperative (within 14 days)^b^** | | |  | **Postoperative (within 14-180 days)^c^** | | |
| **CEA & SCC ^a^** | **N (%)** | **Adjusted HR (95% CI)^d^** | ***P* value** |  | **N (%)** | **Adjusted HR (95% CI)^d^** | ***P* value** |
| Negative-Negative | 245 (64.5) | Ref. |  |  | 159 (76.8) | Ref. |  |
| Negative-Positive | 119 (31.3) | 1.53 (0.94-2.47) | 0.085 |  | 39 (18.8) | 2.06 (1.13-3.73) | 0.018 |
| Positive-Negative | 9 (2.4) | 1.71 (0.40-7.38) | 0.471 |  | 7 (3.4) | 1.41 (0.29-6.82) | 0.666 |
| Positive-Positive | 7 (1.8) | 3.71 (1.28-10.76) | 0.016 |  | 2 (1.0) | 2.46 (0.30-20.42) | 0.404 |
| *P* for trend^e^ |  |  | 0.007 |  |  |  | 0.031 |
| **Abbreviations:** CEA, Carcinoembryonic antigen; CI, confidence interval; HR, hazard ratio; N, number of patients available; Ref., reference category; SCC, Squamous cell carcinoma associated antigen. | | | | | | | |
| ^a^The groups were divided into three classes according to serum concentrations of the CEA and SCC: Negative-Negative, serum concentration of SCC < 1.5 ng/mL and CEA < 5 ng/mL; Negative-Positive, serum concentration of SCC < 1.5 ng/mL and CEA ≥ 5 ng/mL, Positive-Negative, serum concentration of the SCC ≥ 1.5 ng/mL and CEA < 5 ng/mL; Positive-Positive, serum concentration of SCC ≥1.5 ng/mL and CEA ≥ 5 ng/mL. ^b^The concentrations of tumor markers adopted were tested latest within 14 days before the radical resection.  ^c^The concentrations of tumor markers were tested firstly in 14-180 days after the radical resection.  ^d^HRs were adjusted for a set of fixed confounders including age, sex, smoking history, alcohol-use history, tumor site, TNM stage, number of lymph nodes harvested, tumor size, surgical margin status, preoperative neoadjuvant treatment, and postoperative adjuvant treatment.  ^e^*P* for trend was derived by treating the ordinal variable as a continuous variable. | | | | | | | |

| **Supplementary** **Table 2. The subgroup analysis of CEA and SCC stratified by TNM stage in ESCC patients receiving radical resection using multivariable Cox proportional hazards regression models** | | | | | | | |
| --- | --- | --- | --- | --- | --- | --- | --- |
|  | |  | **Preoperative (within 14 d)^b^** | |  | **Postoperative (within 14-180 d)^c^** | |
| **Tumor Markers** | | **Stage** | **N (%)** | **Adjusted HR (95% CI)^d^** |  | **N (%)** | **Adjusted HR (95% CI)^d^** |
| **CEA**^a^ |  |  |  |  |  |  |  |
|  | Negative (< 5.0 ng/mL) | Stage I-II | 763 (96.2) | Ref. |  | 247 (97.2) | Ref. |
|  | Positive (≥ 5.0 ng/mL) |  | 30 (3.8) | 1.35 (0.95-1.92) |  | 7 (2.8) | 4.54 (1.54-13.42) |
|  | Negative (< 5.0 ng/mL) | Stage III | 370 (97.9) | Ref. |  | 305 (97.8) | Ref. |
|  | Positive (≥ 5.0 ng/mL) |  | 8 (2.1) | 0.58 (0.14-2.38) |  | 7 (2.2) | 1.68 (0.61-4.58) |
| **SCC^a^** |  |  |  |  |  |  |  |
|  | Negative (< 1.5 ng/mL) | Stage I-II | 260 (73.9) | Ref. |  | 127 (85.2) | Ref. |
|  | Positive (≥ 1.5 ng/mL) |  | 92 (26.1) | 1.56 (0.83-2.94) |  | 22 (24.8) | 4.05 (1.81-9.07) |
|  | Negative (< 1.5 ng/mL) | Stage III | 98 (57.3) | Ref. |  | 134 (76.1) | Ref. |
|  | Positive (≥ 1.5 ng/mL) |  | 73 (42.7) | 1.11 (0.69-1.79) |  | 42 (23.9) | 2.05 (1.20-3.51) |
| **Abbreviations:** CEA, Carcinoembryonic antigen; CI, confidence interval; HR, hazard ratio; N, number of patients available; Ref., reference category; SCC, Squamous cell carcinoma associated antigen. | | | | | | | |
| ^a^The cutoff values refer to the recommended upper limit of the normal range used in clinical practice.  ^b^The concentrations of tumor markers adopted were tested latest within 14 days before the radical resection.  ^c^The concentrations of tumor markers were firstly tested in 14-180 days after the radical resection.  ^d^HRs were adjusted for a set of fixed confounders including age, sex, smoking history, alcohol-use history, tumor site, TNM stage, number of lymph nodes harvested, tumor size, surgical margin status, preoperative neoadjuvant treatment, and postoperative adjuvant treatment. | | | | | | | |

| **Supplementary Table 3. The multivariable Cox proportional hazards regression model of postoperative SCC** | | |
| --- | --- | --- |
| **Variables** | **Adjusted HR** | ***P*-value** |
| **Postoperative SCC^a^** |  |  |
| Negative (< 1.5 ng/mL)^b^ | Reference |  |
| Positive (≥ 1.5 ng/mL)^b^ | 2.67 (1.70-4.17) | <0.001 |
| **Gender** |  |  |
| Female | Reference |  |
| Male | 0.88 (0.47-1.65) | 0.697 |
| **Age** |  |  |
| <65 | Reference |  |
| ≥65 | 1.69 (1.11-2.57) | 0.015 |
| **Tumor Site** |  |  |
| Upper | Reference |  |
| Middle | 0.75 (0.42-1.34) | 0.329 |
| Lower | 0.94 (0.48-1.83) | 0.850 |
| **Lymph Nodes** |  |  |
| <20 | Reference |  |
| ≥20 | 1.17 (0.74-1.85) | 0.501 |
| **TNM Stage** |  |  |
| I-II | Reference |  |
| III | 2.35 (1.48-3.72) | <0.001 |
| **Tumor Size (cm)** |  |  |
|  | 1.06 (0.97-1.16) | 0.173 |
| **Surgical Margin Status** |  |  |
| Negative | Reference |  |
| Positive | 0.91 (0.12-6.89) | 0.928 |
| **Preoperative Neoadjuvant Treatment** | |  |
| No | Reference |  |
| Yes | 0.89 (0.49-1.63) | 0.713 |
| **Postoperative Adjuvant Treatment** | |  |
| No (Surgery only) | Reference |  |
| Yes | 0.69 (0.36-1.32) | 0.268 |
| **Smoking History** | |  |
| No | Reference |  |
| Yes | 0.95 (0.52-1.75) | 0.875 |
| **Alcohol-Use History** | |  |
| No | Reference |  |
| Yes | 1.13 (0.66-1.95) | 0.649 |
| ^a^The concentrations of tumor markers adopted were tested firstly within 14-180 days before the radical resection.  ^b^The cutoff values refer to the recommended upper limit of the normal range used in clinical practice. | | |

| **Supplementary Table 4. The multivariable Cox proportional hazards regression model of preoperative SCC** | | |
| --- | --- | --- |
| **Variables** | **Adjusted HR** | ***P*-value** |
| **Preoperative SCC^a^** |  |  |
| Negative (< 1.5 ng/mL)^b^ | Reference |  |
| Positive (≥ 1.5 ng/mL)^b^ | 1.32 (0.89-1.95) | 0.161 |
| **Gender** |  |  |
| Female | Reference |  |
| Male | 0.84 (0.47-1.52) | 0.57 |
| **Age** |  |  |
| <65 | Reference |  |
| ≥65 | 1.58 (1.09-2.31) | 0.017 |
| **Tumor Site** |  |  |
| Upper | Reference |  |
| Middle | 0.39 (0.21-0.71) | 0.002 |
| Lower | 0.5 (0.3-0.84) | 0.009 |
| **Lymph Nodes** |  |  |
| <20 | Reference |  |
| ≥20 | 0.67 (0.44-1.02) | 0.065 |
| **TNM Stage** |  |  |
| I-II | Reference |  |
| III | 4.81 (3.16-7.31) | <0.001 |
| **Tumor Size (cm)** |  |  |
|  | 1.09 (0.97-1.23) | 0.148 |
| **Surgical Margin Status** |  |  |
| Negative | Reference |  |
| Positive | 0.36 (0.08-1.58) | 0.174 |
| **Preoperative Neoadjuvant Treatment** | |  |
| No | Reference |  |
| Yes | 0.91 (0.49-1.7) | 0.76 |
| **Postoperative Adjuvant Treatment** | |  |
| No (Surgery only) | Reference |  |
| Yes | 0.94 (0.64-1.38) | 0.745 |
| **Smoking History** |  |  |
| No | Reference |  |
| Yes | 1.24 (0.69-2.23) | 0.468 |
| **Alcohol-Use History** |  |  |
| No | Reference |  |
| Yes | 1.15 (0.69-1.92) | 0.589 |
| ^a^The concentrations of tumor markers adopted were tested latest within 14 days before the radical resection.  ^b^The cutoff values refer to the recommended upper limit of the normal range used in clinical practice. | | |

| **Supplementary Table 5. The multivariable Cox proportional hazards regression model of Postoperative CEA** | | |
| --- | --- | --- |
| **Variables** | **Adjusted HR** | ***P*-value** |
| **Postoperative CEA^a^** |  |  |
| Negative (< 5 ng/mL)^b^ | Reference |  |
| Positive (≥ 5 ng/mL)^b^ | 2.36 (1.14-4.86) | 0.015 |
| **Gender** |  |  |
| Female | Reference |  |
| Male | 1.13 (0.74-1.73) | 0.563 |
| **Age** |  |  |
| <65 | Reference |  |
| ≥65 | 1.52 (1.14-2.02) | 0.005 |
| **Tumor Site** |  |  |
| Upper | Reference |  |
| Middle | 0.63 (0.42-0.92) | 0.018 |
| Lower | 0.84 (0.53-1.32) | 0.443 |
| **Lymph Nodes** |  |  |
| <20 | Reference |  |
| ≥20 | 0.92 (0.67-1.26) | 0.596 |
| **TNM Stage** |  |  |
| I-II | Reference |  |
| III | 2.21 (1.6-3.05) | <0.001 |
| **Tumor Size (cm)** |  |  |
|  | 1.1 (1.01-1.19) | 0.023 |
| **Surgical Margin Status** |  |  |
| Negative | Reference |  |
| Positive | 0.50 (0.12-2.04) | 0.336 |
| **Preoperative Neoadjuvant Treatment** | |  |
| No | Reference |  |
| Yes | 1.25 (0.82-1.92) | 0.302 |
| **Postoperative Adjuvant Treatment** | |  |
| No (Surgery Only) | Reference |  |
| Yes | 0.71 (0.39-1.31) | 0.274 |
| **Smoking History** | |  |
| No | Reference |  |
| Yes | 0.97 (0.65-1.45) | 0.877 |
| **Alcohol-Use History** | |  |
| No | Reference |  |
| Yes | 0.96 (0.66-1.40) | 0.752 |
| ^a^The concentrations of tumor markers adopted were tested firstly within 14-180 days before the radical resection.  ^b^The cutoff values refer to the recommended upper limit of the normal range used in clinical practice. | | |

| **Supplementary Table 6. The multivariable Cox proportional hazards regression model of preoperative CEA** | | |
| --- | --- | --- |
| **Variables** | **Adjusted HR** | ***P*-value** |
| **Preoperative CEA^a^** |  |  |
| Negative (< 5 ng/mL)^b^ | Reference |  |
| Positive (≥ 5 ng/mL)^b^ | 1.30 (0.69-2.46) | 0.407 |
| **Gender** |  |  |
| Female | Reference |  |
| Male | 1.00 (0.71-1.40) | 0.980 |
| **Age** |  |  |
| <65 | Reference |  |
| ≥65 | 1.42 (1.12-1.78) | 0.003 |
| **Tumor Site** |  |  |
| Upper | Reference |  |
| Middle | 0.55 (0.41-0.72) | <0.001 |
| Lower | 0.54 (0.37-0.78) | <0.001 |
| **Lymph Nodes** |  |  |
| <20 | Reference |  |
| ≥20 | 0.85 (0.66-1.1) | 0.219 |
| **TNM Stage** |  |  |
| I-II | Reference |  |
| III | 3.31 (2.58-4.24) | <0.001 |
| **Tumor Size (cm)** |  |  |
|  | 1.11 (1.04-1.19) | 0.002 |
| **Surgical Margin Status** |  |  |
| Negative | Reference |  |
| Positive | 0.77 (0.33-1.76) | 0.530 |
| **Preoperative Neoadjuvant Treatment** | |  |
| No | Reference |  |
| Yes | 1.21 (0.75-1.96) | 0.426 |
| **Postoperative Adjuvant Treatment** | |  |
| No (Surgery only) | Reference |  |
| Yes | 0.95 (0.75-1.21) | 0.689 |
| **Smoking History** |  |  |
| No | Reference |  |
| Yes | 1.10 (0.79-1.53) | 0.586 |
| **Alcohol-Use History** |  |  |
| No | Reference |  |
| Yes | 1.00 (0.74-1.36) | 0.983 |
| ^a^The concentrations of tumor markers adopted were tested latest within 14 days before the radical resection.  ^b^The cutoff values refer to the recommended upper limit of the normal range used in clinical practice. | | |

| **Supplementary Table 7. The multivariable Cox proportional hazards regression model of preoperative SCC and CEA** | | |
| --- | --- | --- |
| **Variables** | **Adjusted HR** | ***P*-value** |
| **Preoperative CEA^a^** |  |  |
| Negative (< 5 ng/mL)^b^ | Reference |  |
| Positive (≥ 5 ng/mL)^b^ | 2.14 (0.91-5.05) | 0.083 |
| **Preoperative SCC^a^** |  |  |
| Negative (< 1.5 ng/mL)^b^ | Reference |  |
| Positive (≥ 1.5 ng/mL)^b^ | 1.58 (0.99-2.5) | 0.053 |
| **Gender** |  |  |
| Female | Reference |  |
| Male | 0.8 (0.41-1.56) | 0.51 |
| **Age** |  |  |
| <65 | Reference |  |
| ≥65 | 1.38 (0.89-2.14) | 0.145 |
| **Tumor Site** |  |  |
| Upper | Reference |  |
| Middle | 0.45 (0.25-0.8) | 0.007 |
| Lower | 0.3 (0.14-0.63) | 0.002 |
| **Lymph Nodes** |  |  |
| <20 | Reference |  |
| ≥20 | 0.66 (0.4-1.1) | 0.11 |
| **TNM Stage** |  |  |
| I-II | Reference |  |
| III | 4.58 (2.79-7.51) | <0.001 |
| **Tumor Size (cm)** |  |  |
|  | 1.11 (0.97-1.26) | 0.119 |
| **Surgical Margin Status** |  |  |
| Negative | Reference |  |
| Positive | 0.55 (0.12-2.54) | 0.443 |
| **Preoperative Neoadjuvant Treatment** | |  |
| No | Reference |  |
| Yes | 1.06 (0.5-2.24) | 0.879 |
| **Postoperative Adjuvant Treatment** | |  |
| No (Surgery Only) | Reference |  |
| Yes | 0.84 (0.53-1.33) | 0.457 |
| **Smoking History** |  |  |
| No | Reference |  |
| Yes | 1.7 (0.85-3.42) | 0.135 |
| **Alcohol-Use History** |  |  |
| No | Reference |  |
| Yes | 0.98 (0.53-1.8) | 0.942 |
| ^a^The concentrations of tumor markers adopted were tested firstly within 14-180 days before the radical resection.  ^b^The cutoff values refer to the recommended upper limit of the normal range used in clinical practice. | | |

| **Supplementary Table 7. The multivariable Cox proportional hazards regression model of postoperative SCC and CEA** | | |
| --- | --- | --- |
| **Variables** | **Adjusted HR** | ***P*-value** |
| **Postoperative CEA^a^** |  |  |
| Negative (< 5 ng/mL)^b^ | Reference |  |
| Positive (≥ 5 ng/mL)^b^ | 1.34 (0.37-4.87) | 0.656 |
| **Postoperative SCC^a^** |  |  |
| Negative (< 1.5 ng/mL)^b^ | Reference |  |
| Positive (≥ 1.5 ng/mL^)b^ | 2.05 (1.15-3.65) | 0.015 |
| **Gender** |  |  |
| Female | Reference |  |
| Male | 0.78 (0.45-1.34) | 0.366 |
| **Age** |  |  |
| <65 | Reference |  |
| ≥65 | 1.62 (0.97-2.69) | 0.066 |
| **Tumor Site** |  |  |
| Upper | Reference |  |
| Middle | 0.7 (0.33-1.48) | 0.351 |
| Lower | 1.1 (0.48-2.56) | 0.819 |
| **Lymph Nodes** |  |  |
| <20 | Reference |  |
| ≥20 | 1.34 (0.76-2.37) | 0.307 |
| **TNM Stage** |  |  |
| I-II | Reference |  |
| III | 1.93 (1.12-3.34) | 0.018 |
| **Tumor Size (cm)** |  |  |
|  | 1.13 (1.03-1.23) | 0.011 |
| **Surgical Marginal** |  |  |
| Negative | Reference |  |
| Positive | 1.59 (0.19-13.38) | 0.670 |
| **Preoperative neoadjuvant treatment** | |  |
| No | Reference |  |
| Yes | 1 (0.42-2.42) | 0.993 |
| **Postoperative adjuvant treatment** | |  |
| No (Surgery only) | Reference |  |
| Yes | 0.59 (0.24-1.46) | 0.254 |
| **Smoking history** |  |  |
| No | Reference |  |
| Yes | 1.07 (0.45-2.54) | 0.872 |
| **Alcohol-use history** |  |  |
| No | Reference |  |
| Yes | 1.37 (0.58-3.19) | 0.473 |
| ^a^The concentrations of tumor markers adopted were tested latest within 14 days before the radical resection.  ^b^The cutoff values refer to the recommended upper limit of the normal range used in clinical practice. | | |

| **Supplementary Table 8: The independent test of SCC and CEA** | | | | | | |
| --- | --- | --- | --- | --- | --- | --- |
| **Preoperative** | | |  | **Postoperative** |  |  |
| **CEA SCC** | Negative | Positive |  | **CEA SCC** | Negative | Positive |
| Negative | 245 | 9 |  | Negative | 159 | 7 |
| Positive | 119 | 7 |  | Positive | 39 | 2 |
| Chi square test: *p value*= 0.517 | | |  | Fisher exact test: *p value*= 0.990 | | |
